# Change in Care Dependency and Nursing Care Problems in Nursing Home Residents with and without Dementia: A 2-Year Panel Study

**DOI:** 10.1371/journal.pone.0141653

**Published:** 2015-10-29

**Authors:** Sandra Schüssler, Christa Lohrmann

**Affiliations:** Institute of Nursing Science, Medical University of Graz, Austria; University of Brescia, ITALY

## Abstract

Over time, chronic conditions like dementia can lead to care dependency and nursing care problems, often necessitating nursing home admission. This panel study (2012–2014) aims to explore changes in care dependency and nursing care problems (incontinence, malnutrition, decubitus, falls and restraints) in residents with and without dementia over time. In total, nine Austrian nursing homes participated, including 258 residents (178 with, 80 without dementia) who completed all five measurements. Data were collected with the International Prevalence Measurement of Care Problems questionnaire, the Care Dependency Scale and the Mini-Mental State Examination-2. Repeated measures ANOVA and crosstabs were used to analyse changes. The results showed that care dependency in dementia residents increased significantly for all 15 items of the Care Dependency Scale, with the highest increase being residents’ day-/night pattern, contact with others, sense of rules/values and communication. In contrast, care dependency in residents without dementia increased for four of the 15 items, with the highest increase being for continence, followed by getting (un)dressed. With respect to the assessed nursing care problems, residents with dementia and those without only differed significantly in terms of an increase in urinary- (12.3% vs. 14.2%), fecal- (17.4% vs. 10%), and double incontinence (16.7% vs. 11.9%). The results indicated that residents with dementia experienced increased care dependency in different areas than residents without dementia. Furthermore, residents with dementia experienced a lower increase in urinary incontinence but a higher increase in fecal- and double incontinence. These results help professionals to identify areas for improvement in dementia care.

## Introduction

Worldwide, the aging population is growing and people are now living longer than ever before [1]. This transition is related with an increase in chronic conditions like cancer and dementia [1]. As these conditions progress, care dependency and nursing care problems like malnutrition may occur and increase because of declines in physical-, social- and mental abilities [1–3]. Dementia sufferers may be especially affected early in their illness [1].

This study defines care dependency as a process in which an individual’s care demands require professional nursing support because of their decreased ability to provide self-care for physical and psychosocial human needs, like eating and drinking, dressing, communication and social contacts [4]. Nursing care problems include impairments (e.g., malnutrition, incontinence, falls) and risks related to health or treatment (e.g., restraints) that nursing home residents are not able to address themselves [5].

Care dependency, its progression and consequential nursing care problems are identified ascommon reasons that people relocate to a nursing home [1,6]. In this setting, frequent problems include pressure ulcers, incontinence, falls, malnutrition and restraints [1,7–9]. Therefore many nursing homes today monitor their quality of care [7,8] to stabilize or improve care dependency and/or nursing care problems for residents [7,10,11] and to avoid negative consequences like reduced quality of life, high health care costs and mortality [7,11–13].

Few international studies explore changes in care dependency (in various human needs) and/or nursing care problems over time. Most have only examined changes in residents as a group [14–17], or in residents with/without cognitive impairments [18–20]. Research exploring changes in care dependency (in various human needs) and nursing care problems in residents with diagnosed dementia compared to those without is, to our knowledge, missing in the international literature, as are studies that explore changes in care dependency and various nursing care problems together. This research would be important, because it could help to identify differences in the change of care dependency (in different human needs) and nursing care problems between residents with and without dementia. This knowledge would help to tailor care better to the specific needs and nursing care problems of nursing home residents with dementia.

Our study explores changes in care dependency and common nursing care problems in nursing home residents with and without dementia over time using the following research questions: 1) How does care dependency (in various human needs) change in residents with and without dementia over two years and how does the change differ between these two groups? 2) How do nursing care problems (pressure ulcers, incontinence, malnutrition, falls, restraints) in residents with and without dementia change over two years and how does the change differ between these two groups?

## Methods

### Design

A panel study was conducted from April 2012—April 2014 with five measurement points (baseline assessment [T0] and follow-ups after six- [T1], 12- [T2], 18- [T3] and 24 months [T4]).

### Setting and sample

A convenience sampling was performed. An Austrian governmental database [21] was used to identify 175 nursing homes with ≥50 beds in two federal states. These nursing homes were invited to participate via post and e-mail, nine of which agreed to participate. All residents from said nursing homes (29 wards) were included in the study if they were present at T0 and could understand German. Comatose and dying residents were excluded as were residents in short-term care. The approval of the Medical University of Graz ethics committee (EK: 23–520 ex 10/11) was obtained and written informed consent was acquired from each participating resident or their legal representative.

### Instruments

The following instruments were used by trained nursing staff at all measurement points.

#### Demographic data and prevalence of nursing care problems

Age, gender, medically diagnosed dementia and other diseases (based on ICD-10), length of stay in thenursing home and prevalence of nursing care problems (incontinence, malnutrition, restraints, pressure ulcers and falls) were collected using the Austrian version of the *International Prevalence Measurement of Care Problems* questionnaire [9,11]. This standardized questionnaire originally developed at the Maastricht University had both dichotomous (yes/no) and multiple answer options and included assessment instruments (e.g., Care Dependency Scale) [9] The Dutch questionnaire was forward and backward translated and double-checked for nomenclature and cultural differences by researchers and experts in the field of quality nursing care. An in-depth description of the questionnaire can be found in van Nie-Visser et al [9]. Additionally, the type of dementia and the diagnosing physician were collected by nursing staff using patient medical records.

#### Care dependency

Care dependency was assessed using the German version of the *Care Dependency Scale* (CDS) [22], originally developed in the Netherlands to assess care dependency in residents with dementia [10]. The psychometric properties of the CDS have been well-tested for people with and without dementia and for different settings, like nursing homes and hospitals [10,22]. The CDS contains 15 items (physical and psychosocial human needs) that are assessed by a 5-point Likert scale. A resident can be assigned between 15 and 75 points [10,22]. According to Dijkstra et al. [23], residents with sum scores of 15–24 are classified as *completely care dependent*, 25–44 as *to a great extent care dependent*, 45–59 as *partially care dependent*, 60–69 as *to a limited extent care dependent* and 70–75 as *almost care independent*.

#### Cognitive impairment

Cognitive impairment in residents with and without dementia was assessed using the German version of the *Mini-Mental State Examination 2* (MMSE-2) [24]. This version has the same structure and scoring as the original MMSE. Small changes were made to improve items (e.g., words added for repetition exercises include *milk*, *sensible* and *before*) as a means to standardize translation into other languages and cultures. The English version of the MMSE-2 was forward- and backward translated by professional translators. The 30 items (every item 0 or 1 point) of the MMSE-2 include questions on registration; orientation in time and place; recollection; attention and calculation; naming; repetition; comprehension; reading; writing; and drawing. Lower scores on the MMSE-2 indicate higher cognitive impairment [24]. Persons are categorized as having mild dementia/slight cognitive impairment when they reach 24–20 points on the scale, moderate dementia/moderate cognitive impairment at 19–10 points and severe dementia/severe cognitive impairment at below 10 points [25].

### Data collection

Data were collected in each nursing home at T0 and during four follow-ups (see design part) with a one month period set aside for each data collection. The first author trained all nursing professionals who performed data collection. To ensure objectivity, each resident was assessed by two internal nursing professionals (one involved in the resident’s daily care and a second who was not). The first author visited all nursing home wards on the first day of T0 to ensure data were collected properly and clarify any ambiguities. After each data collection, the nursing homes sent questionnaires to the researchers.

### Statistical analysis

Statistical analyses were performed using IBM^®^ SPSS^®^ Statistics, version 22 (IBM Corporation, NY). Only residents who had completed all measurement points were included in the analysis. Sample characteristics were analyzed using means and standard deviations for age, length of stay and MMSE-2 as metric variables. Differences between residents with and without dementia at T0 were explored using unpaired t-tests. Gender, medical diseases/disorders and bedridden status were analyzed as categorical variables using crosstabs; differences between the groups at T0 were explored using chi-squared tests. The change of the MMSE-2 sum score, the overall CDS-sum score and the individual 15 CDS items were first analyzed for residents with and without dementia using one-factorial ANOVA with repeated measures and then between residents with and without dementia using a two-factorial ANOVA with repeated measures on one factor. The changes in medical diseases/disorders, bedridden status and nursing care problems were analyzed using crosstabs and differences (T0-T4) within and between the groups were explored using McNemar tests. In all analyses, p-values ≤ .05 were considered to be significant.

## Results

### Sample characteristics

From a total of 815 residents, 527 (65%) participated in the study at T0. Of these residents, 258 completed all measurements points. The response rate (T1, T2, T3, T4) was between 79.6%–88.5%. The most common reason for non-participation (T1-T4) was either that residents had died (63.7%) or refused further participation (14.4%).

Overall, 69% (178 of 258) of the residents had dementia, of which 52% had Alzheimer’s, 15.8% vascular dementia, 19.2% other types of dementia and 13% no specific type of dementia. Diagnoses were mainly made by a neurologist (86.4%).

Table 1 shows significant differences between residents with and without dementia at T0. Residents with dementia had shorter nursing home stays and were less often affected by motor diseases and cancer. Cognitive impairment (MMSE-2) was higher in residents with dementia. The other characteristics did not significantly differ between the two groups.

**Table 1:** Sample characteristics of residents with and without dementia at baseline

Sample characteristics	T0	T0	P-value
	Dementia (n = 178)	No Dementia (n = 80)	
Age (in years), mean ± SD	83.5 ± 7.6	82.0 ± 10.4	.277
Length of stay in nursing home (in years), mean ± SD	2.5±2.8	3.5±3.4	.017
Female, %	83.1	78.8	.397
Medical diseases/disorders, %			
Cardio vascular disease	56.5	53.8	.681
Motor disorder/disease	29.4	42.5	.039
Diabetes mellitus	25.4	17.5	.162
Depression	22.6	26.3	.524
Eye/ear disorder	16.9	23.8	.199
Disorder/disease of the digestive tract, including intestinal obstruction, peritonitis, hernia, liver, gallbladder, pancreas	21.5	12.5	.088
Disorder/Disease of kidney/urinary tract, sexual organs	20.9	12.5	.107
CVA/hemiparesis	16.4	12.5	.422
Endocrine-nutritional or metabolic illness/disease	13.0	15.0	.664
Cancer	2.8	11.3	.014
Nervous system disorder, excluding CVA	11.9	8.8	.458
Respiratory disorder/disease, including nose and tonsils	6.2	1.3	.111
Disease of blood or blood related organs	1.7	2.5	.648
Bedridden,%	1.7	2.5	1.000
MMSE-2, mean sumscore ± SD	16.5 ± 9.2	25.9 ± 4.6	< .001

Over two years (T0-T4), residents with dementia compared to residents without experienced a significant greater increase in motor diseases (15.8% vs 5%; P = < .001) and cognitive impairment assessed with the MMSE-2 (-3.8 points vs -0.9 points; P = .001). The change of other medical diseases/disorders and bedridden status did not significantly differ between the two groups.

### Change in care dependency

**Fig 1:**
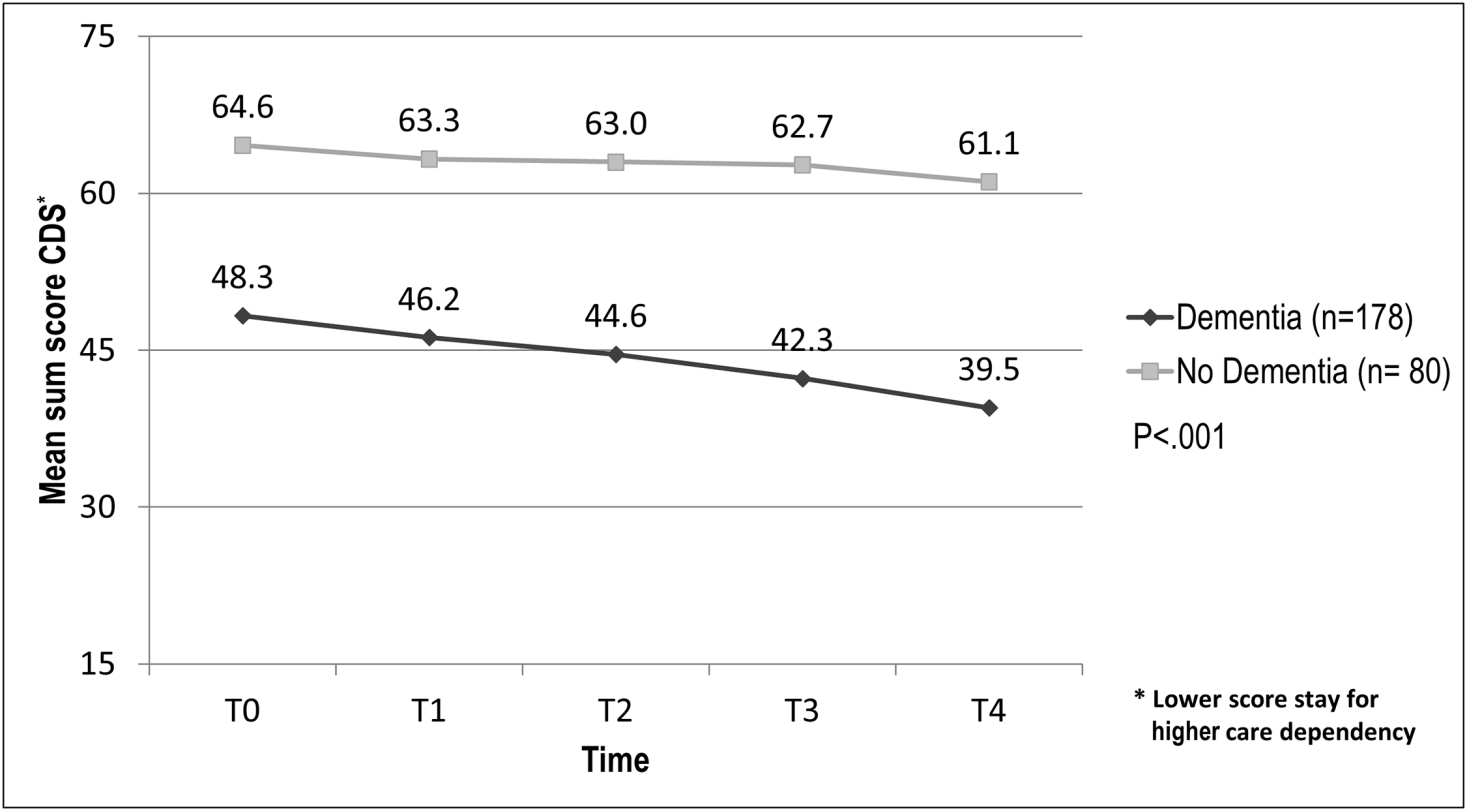
Change in care dependency in residents with and without dementia over time .

Over two years, care dependency increased significantly in residents with (P < .001) and without dementia (P = .04). A comparison of these two groups revealed that the increase in care dependency was significantly higher in residents with dementia (-8.8 vs. -3.5 points; p < .001) (Fig 1).

**Fig 2:**
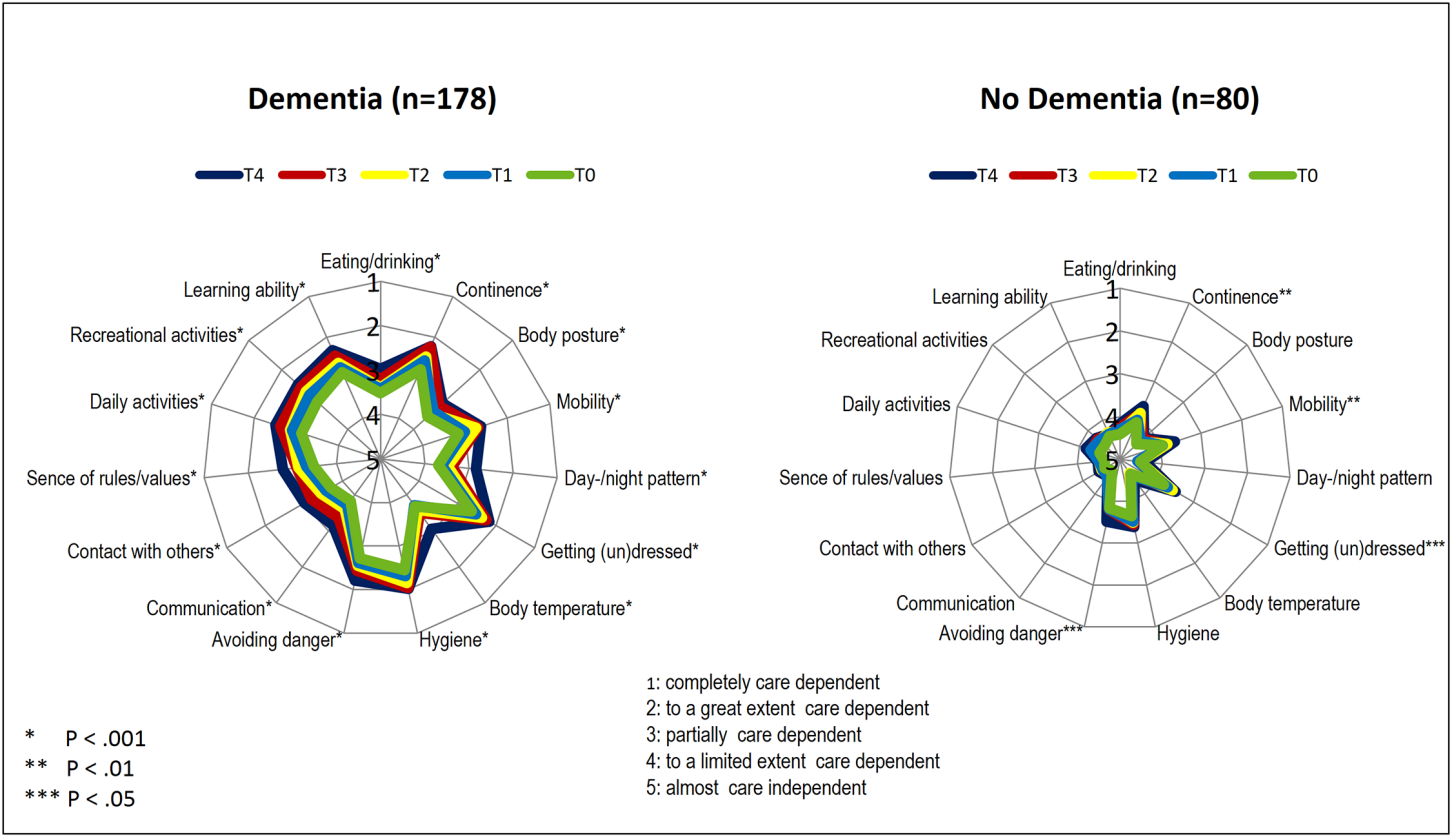
Change in care dependency on an item level in residents with and without dementia over time.

Fig 2 highlights that over two years, care dependency for all 15 CDS items increased significantly in residents with dementia. Residents without dementia became increasingly care dependent for 4 of the 15 items.

The comparison of the 15 CDS items between residents with and without dementia showed that over two years, residents with dementia experienced a significantly higher increase in care dependency for the items day-/night pattern, body temperature, communication, contact with others, sense of rules and values and learning ability. The other items did not differ significantly between the groups (Table 2).

**Table 2:** Mean differences in the change of care dependency on an item level between residents with and without dementia.

15 CDS Items	T0 to T4	T0 to T4	P-value
	Dementia (n = 178)	No Dementia (n = 80)	
	Mean Difference ± SD	Mean Difference ± SD	
**Eating/drinking**	-0.54±1.08	-0.24±1.02	.074
Ability to eat and drink/ prepare food/beverages			
**Continence**	-0.56 ± 1.21	-0.41 ± 1.22	.193
Ability to voluntarily control the discharge of urine/bowel movements and to take appropriate measures in response			
**Body posture**	-0.45 ± 1.24	-0.23 ± 1.01	.462
Ability to adopt appropriate activity-dependent positions			
**Mobility**	-0.54 ± 1.27	-0.31 ± 1.00	.064
Ability to move independently and without assistance			
**Day-/night pattern**	-0.85 ± 1.28	-0.13 ± 0.99	< .001
Ability to independently maintain an appropriate day/night cycle			
**Getting (un)dressed**	-0.46 ± 1.08	-0.33 ± 1.08	.470
Ability to get (un)dressed without assistance			
**Body temperature**	-0.45 ± 1.24	-0.23 ± 1.01	.462
Ability to maintain body temperature despite external influences			
**Hygiene**	-0.43 ± 1.02	-0.26 ± 0.95	.254
Ability to maintain personal hygiene and grooming			
**Avoiding danger**	-0.52 ± 1.15	-0.30 ± 1.26	.346
Ability to recognize danger and ensure safety			
**Communication**	-0.67 ± 1.23	-0.16 ± 0.65	< .001
Ability to communicate with others (verbally and non-verbally)			
**Contact with others**	-0.70 ± 1.24	-0.16 ± 0.97	.001
Ability to make, maintain and end social contact			
**Sense of rules/values**	-0.70 ± 1.23	-0.16± 0.68	.002
Ability to observe rules and values and assert the protection of privacy			
**Daily activities**	-0.61 ± 1.27	-0.34 ± 1.07	.095
Ability to manage/structure/perform daily activities unaided (e.g. household, shopping, money, appointments)			
**Recreational activities**	-0.58 ± 1.22	-0.26 ± 0.99	.148
Ability to make sensible use of free time/participate in leisure activities unaided			
**Learning ability**	-0.55 ± 1.28	-0.05 ± 1.10	.003
Ability to acquire knowledge/skills and/or to retain knowledge/skills learned in the past			

### Change in nursing care problems

**Fig 3:**
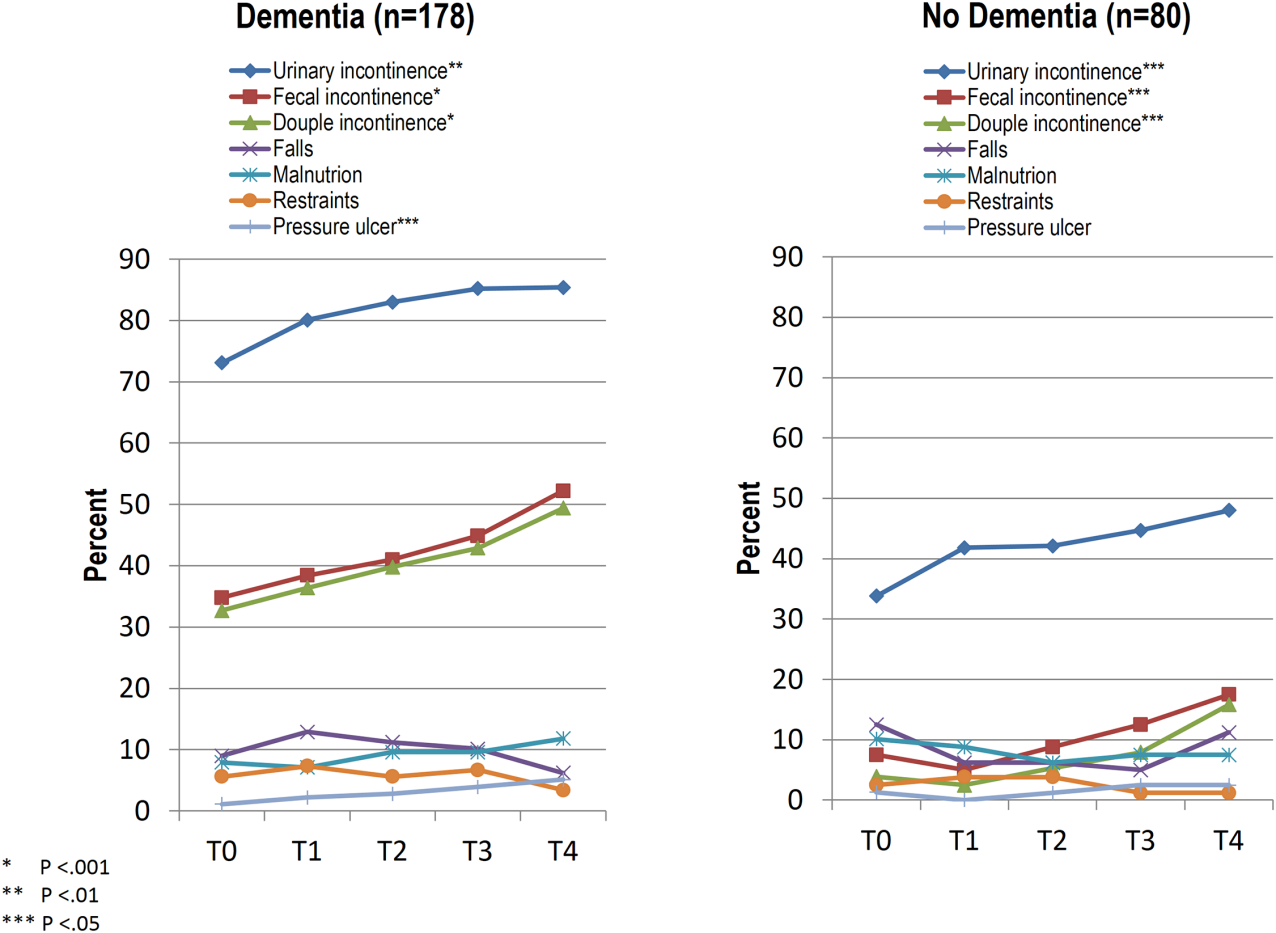
Change in nursing care problems in residents with and without dementia over time

Fig 3 indicates that over two years, the number of residents with dementia who experienced fecal incontinence increased by 17.4% (from 34.8%–52.2%), followed by double incontinence with 16.7% (from 32.7%–49.4%), urinary incontinence with 12.3% (from 73.1%–85.4%) and pressure ulcers with 4% (from 1.1%–5.1%). In residents without dementia, urinary incontinence increased by 14.2% (from 33.8%–48.0%), followed by double incontinence with 11.9% (from 3.9%–15.8%) and fecal incontinence with 10% (from 7.5%–17.5%). No other nursing care problems changed significantly for residents with and without dementia.

The comparison of nursing care problems between residents with and without dementia revealed that over two years, residents with dementia experienced a significant lower increase in urinary incontinence (12.3% vs 14.2%, P < .001), but a significant higher increase in fecal- and double incontinence (fecal: 17.4% vs 10%, P < .001; double: 16.7% vs 11.9, P < .001). The changes in all other nursing care problems did not differ significantly between residents with and without dementia.

## Discussion

### Care dependency

Results showed that care dependency in residents with and without dementia increased significantly over two years, but for residents with dementia, it increased significantly more than in residents without dementia.

The fact that both groups changed significantly confirmed the understanding that once dependency in older people with chronic diseases takes hold, it tends to become chronic and progressive [1]. In our study, both groups were already care dependent at baseline, but more so for residents with dementia. Despite this fact, they experienced significantly higher care dependency over time. This may be because dementia is known to be the greatest independent contributor to care dependency based on its association with early physical, mental and behavioral decline [1]. In our study, residents with dementia were in a moderate stage of their illness at baseline (MMSE-2: sum score 16.5).

Dijkstra et al. [10] used the CDS scale to explore changes in care dependency in residents with Alzheimer’s after two years and found a higher increase of care dependency (-11.37 points) than in our study, despite the fact that their residents were, at baseline, more care dependent. The higher increase of care dependency may be because they only included residents with Alzheimer’s, which is characterized by its gradual progression. Other types of dementia, like vascular dementia, can progress differently [26].

Our results highlighted that care dependency in all 15 items of the CDS significantly increased for residents with dementia; especially in terms of day-/night pattern, contact with others, sense of rules and values and communication. Dijkstra et al. [10] also identified, with the exception of mobility, a significant increase in care dependency in the individual CDS items. Furthermore, they highlighted that dependency, specifically in terms of contact with others and communication, as well as the degree of care dependency, is a strong predictor that residents with dementia will become increasingly care dependent over time. In the present study, care dependency in residents without dementia only increased significantly in the areas of continence, mobility, getting (un)dressed and avoiding danger. This indicates that care dependency in residents without dementia only increased in physical areas in contrast to residents with dementia who experienced great increases in psychosocial areas.

### Nursing care problems

This study highlights that over two years, residents with and without dementia experienced significantly more incontinence. Urinary incontinence increased more in residents without dementia, while fecal- and double incontinence increased more in residents with dementia. The other nursing care problems, except for pressure ulcers, did not change significantly within and between the two groups; the prevalence of other problems was definitively lower compared to incontinence.

The significant increase in incontinence in residents with and without dementia may be related to a general increase in care dependency, which is an important risk factor for older people developing urinary or fecal incontinence [16,27–29].

The literature shows that impairment of toilet use is one of the highest risk factors for urinary-and fecal incontinence in nursing home residents (odds ratio: 5.6 and. 7.4, respectively) [28,30]. In accordance with our results, care dependency in residents with and without dementia increased significantly in the area of continence. For this CDS item, residents without dementia experienced the highest increase in care dependency over time compared to the other 14 items.

The literature identifies further risk factors in specific areas of care dependency as eating, dressing, hygiene and mobility [28,30]. In the present study, care dependency in these risk factors increased significantly in residents with dementia, but it is possible that additional areas of care dependency (e.g., learning ability, communication) influenced the increase of incontinence in dementia residents. Such psychosocial areas are often not included in instruments (e.g., Katz Index, Barthel Index) that measure care dependency. The increase of incontinence in residents without dementia may also be influenced by increasing care dependency in mobility, getting (un)dressed and avoiding danger. Dependency in the ability to avoid danger (e.g., falls) might be connected with mobility problems and could necessitate bathroom accompaniment; should this not be executed in a timely way, incontinence could be reinforced. Care dependency in eating/drinking and hygiene did not increase significantly in residents without dementia and may have had a weaker influence on the increase of incontinence than the other areas in our study.

Besides care dependency, the international literature describes many other risk factors for incontinence. These include aging-related anatomic and physiological changes in the anorectal area and/or in the lower urinary tract, diabetes, (increasing) cognitive impairment, moderate to severe dementia, stroke, depression, pressure ulcers and physical restraints [1, 25, 28–31]. With respect to the medical diseases/disorders assessed as well as cognition and nursing care problems, only residents with dementia displayed significant changes over time in terms of increases in motor disorders/diseases and pressure ulcers. These areas, and the fact that on average residents with dementia were in the moderate stage of the illness with significantly increasing cognitive impairment and care dependency, may have influenced the increase in incontinence.

The fact that residents with dementia experienced a significantly lower increase in urinary incontinence may be explained by their higher prevalence of urinary incontinence (73.1% vs 33.8%) at baseline. This may be, besides the other described risk factors, a reason why residents with dementia exhibited a greater increase of double incontinence than those without dementia, because urinary incontinence is a risk factor for developing fecal incontinence [27,29].

Our study does have some limitations. The participation of nine nursing homes provides only a glimpse into changes in care dependency and nursing care problems in residents with and without dementia in Austria. Nursing home participation was voluntary. The reasons behind their participation/non-participation were not known by the researchers, leaving open the possibility of a selection bias. This study also provided further no information about behavioral problems, pain and psychotropic medications, which are also relevant in nursing homes. Furthermore, data for different types and stages of dementia were not analyzed, which would be important to better understand when developing type- and stage appropriate dementia care.

## Conclusion

Residents with dementia experience a significantly greater increase in care dependency than other residents, primarily in day-/night patterns and psychosocial areas. Care dependency in residents without dementia only increased significantly in physical areas, with the highest increase being in continence. With regard to nursing care problems, the residents with and without dementia only differed significantly in terms of changes in incontinence (urinary, fecal, double).

Because of the high prevalence of and increase in incontinence, particularly in residents with dementia, the authors suggest that clinical practice increase its focus on continence care in nursing homes. A priority should be the training and support of residents in managing their continence according to their needs. Further research should examine changes in care dependency and nursing care problems throughout different stages and types of dementia to gain further knowledge about dementia-specific care in nursing homes.
